# Cocaine Intoxication and Thyroid Storm: Similarity in Presentation and Implications for Treatment

**DOI:** 10.1177/2324709614554836

**Published:** 2014-10-13

**Authors:** Mary E. Lacy, Kristina M. Utzschneider

**Affiliations:** 1 University of Washington, Seattle, WA, USA; 2 VA Puget Sound Health Care System, Seattle, WA, USA

**Keywords:** thyrotoxicosis, Graves’ disease, sympathetic nervous system, clinical decision making

## Abstract

*Introduction*. Cocaine, a widely used sympathomimetic drug, causes thermoregulatory and cardiac manifestations that can mimic a life-threatening thyroid storm. *Case*. A man presented to the emergency department requesting only cocaine detoxification. He reported symptoms over the last few years including weight loss and diarrhea, which he attributed to ongoing cocaine use. On presentation he had an elevated temperature of 39.4°C and a heart rate up to 130 beats per minute. Examination revealed the presence of an enlarged, nontender goiter with bilateral continuous bruits. He was found to have thyrotoxicosis by labs and was treated for thyroid storm and cocaine intoxication concurrently. The patient was ultimately diagnosed with Graves’ disease and treated with iodine-131 therapy. *Conclusion*. Cocaine use should be considered a possible trigger for thyroid storm. Recognition of thyroid storm is critical because of the necessity for targeted therapy and the significant mortality associated with the condition if left untreated.

Thyroid storm is a diagnosis that typically involves an underlying thyrotoxicosis along with a precipitant, such as infection or surgery, with resultant hyperpyrexia and cardiovascular and central nervous system dysfunction.^1^ Cocaine induces a vasoconstrictive and sympathomimetic response^2^ that can clinically mimic thyroid storm. We report the case of a man who presented to the emergency department requesting cocaine detoxification and was found to have concurrent thyroid storm.

## Case Presentation

Mr S presented to a local emergency department requesting cocaine detoxification. He reported a cocaine binge that ended the night prior to admission and regular cocaine use over the past year. He noted a constellation of symptoms, all of which he attributed to cocaine use, including weight loss, diarrhea, mental “fogginess,” diaphoresis, tremor, and palpitations. Past medical history was significant for type 2 diabetes treated with metformin and hypertension treated with lisinopril. His family history was notable for type 1 diabetes in his mother and alcoholism in his father. He had no family history of thyroid abnormalities.

On evaluation, his vital signs were the following: height 170 cm (67 in.), weight 56 kg (124 pounds), rectal temperature of 39.4°C (102.9°F), respiratory rate of 22 breaths per minute, blood pressure of 146/51 mm Hg, and heart rates up to 130 beats per minute. His exam was notable for cachexia, normally sized and reactive pupils, absence of exophthalmos, a diffusely enlarged and nontender thyroid with bilateral continuous bruits, tachycardia with a systolic flow murmur, clear lungs, an intention tremor, and global hyperreflexia. He had significant inattention and distractibility requiring frequent redirection, though he was fully oriented. Laboratory results revealed a thyroid-stimulating hormone (TSH) of <0.01 µIU/mL (normal range = 0.27-4.20), a free T4 of 7.77 ng/dL (0.93-1.70), and a total T3 of 651 ng/dL (85-184). A urine toxicology screen was positive for cocaine and an infectious workup was negative. Chart review confirmed a 9-kg (20-pound) weight loss over the previous 10 months.

The patient was treated concurrently for cocaine intoxication with benzodiazepines and for thyroid storm with hydrocortisone and propylthiouracil followed by saturated solution of potassium iodine. By the following day, his vital signs had normalized and he was notably less distractible. The patient was discharged on methimazole and propranolol. He was referred for outpatient substance abuse treatment.

**Figure 1.**
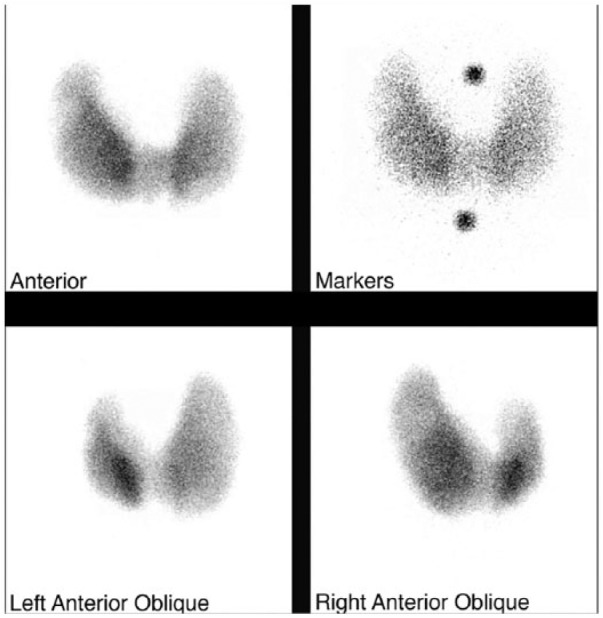
Thyroid uptake and scan. Thyroid scan reflecting uptake of 99mTc-pertechnetate seen from anterior, anterior with markers, left anterior oblique, and right anterior oblique views (clockwise from top left). The upper pole of the right lobe contains an area of photopenia consistent with a cold nodule. He had increased uptake with 4-hour and 24-hour values of 45% (normal = 4% to 20%) and 60% (normal = 10% to 30%), respectively.

On follow-up in endocrine clinic, he had remained cocaine-free. He underwent a cool-off period on continued methimazole and propranolol. Laboratory studies revealed hyperthyroidism but the patient was no longer severely symptomatic and was gaining weight (full results are shown in Table 1). A thyroid uptake and scan 3 months after initial presentation revealed diffusely increased uptake (Figure 1) along with a focus of photopenia within the upper pole of the right lobe consistent with a cold nodule. He was treated with iodine-131 for Graves’ disease immediately following the uptake and scan. He missed his endocrinology appointments postablation and is currently lost to follow-up.

**Table 1.** Composite Vital Signs and Laboratory Values.

	Day 1	Day 2	Day 38
TSH (normal = 0.27-4.20 µIU/mL)	<0.01		<0.01
Free T4 (normal = 0.93-1.70 ng/dL)	7.77	6.89	1.67
Total T3 (normal = 85-184 ng/dL)	651		251
Heart rate (beats per minute)	130	97	81
Temperature (°C)	39.4	37.9	36.7
Weight (kg)	56	57	64

Abbreviations: TSH, thyroid-stimulating hormone; T3, triiodothyronine; T4, thyroxine.

## Discussion

The patient presented with classic signs and symptoms of thyroid storm that could have been attributed solely to his recent cocaine use. A detailed physical exam revealed a symmetrically enlarged thyroid and bruit pathognomonic for Graves’ disease.

This case raises several interesting topics including the similar signs and symptoms of acute cocaine intoxication and thyroid storm, the possible interactions between cocaine and the thyroid, and the optimal treatment of thyroid storm in the setting of acute cocaine ingestion.

## Diagnosis of Thyroid Storm

The diagnosis of thyroid storm is clinical, a combination of patient presentation and laboratory values confirming hyperthyroidism. The degree to which the thyroid hormone levels are elevated does not determine the presence or absence of thyroid storm. Brooks et al showed that serum triiodothyronine (T3) and thyroxine (T4) were similar in patients with uncomplicated thyrotoxicosis and thyroid storm.^3,4^ While patients with thyroid storm did have higher free T4 levels, this laboratory value alone does not establish the presence of life-threatening thyroid storm.^4^ Burch and Wartofsky defined diagnostic criteria for diagnosis of thyroid storm in 1993 to aid clinicians in making the diagnosis and decisions on treatment (see Table 2).^1^

**Table 2.** Diagnostic Criteria for Thyroid Storm^a^.

Thermoregulatory dysfunction (pyrexia); temperature in degrees Celsius		Cardiovascular dysfunction (tachycardia); heart rate in beats per minute	
37.2-37.7	5	90-109	5
37.8-38.2	10	110-119	10
38.3-38.8	15	120-129	15
38.9-39.3	20	130-139	20
39.4-39.9	25	≥140	25
≥40.0	30	Cardiovascular dysfunction (heart failure)	
Central nervous system effects		Absent	0
Absent	0	Mild (pedal edema)	5
Mild (agitation)	10	Moderate (bibasilar rales)	10
Moderate (delirium, psychosis, extreme lethargy)	20	Severe (pulmonary edema)	15
		Cardiovascular dysfunction (atrial fibrillation)	
Severe (seizure, coma)	30	Absent	0
Gastrointestinal-hepatic dysfunction		Present	10
Absent	0		
Moderate (diarrhea, nausea/vomiting, abdominal pain)	10	Precipitant history	
		Negative	0
Severe (unexplained jaundice)	20	Positive	10

a Adapted from Burch and Wartofsky.^1^ Points from each category are added together. Points are calculated to attribute symptoms to thyrotoxicosis when it is not possible to determine true cause of symptoms. A score less than 25 is unlikely to indicate thyroid storm, a score of greater 45 is highly indicative, and a score of 25 to 44 is intermediate.

Cocaine intoxication and thyroid storm lead to clinical pictures with overlapping signs, including hyperpyrexia, tachycardia, and central nervous system disturbances. Because even with early diagnosis and targeted treatment the mortality rate of thyroid storm ranges from 10% to 30%, clinicians should consider assessing for coexisting thyrotoxic state in patients presenting with presumed severe cocaine intoxication.^5^ In the case of Mr S, his score based on diagnostic criteria for thyroid storm (Table 2) was high at 70, and a detailed physical exam revealed a pathologically enlarged thyroid suggesting the need for further laboratory testing, ultimately leading to accurate diagnosis and appropriate treatment.

## Physiology of Thyroid Storm

The mechanisms for the hyperadrenergic state seen in thyroid storm are complex and vary by organ system.^6,7^ Thyrotoxic states have been associated with normal or diminished plasma and urine norepinephrine levels, suggesting the mechanism is not explained by primary adrenergic activation.^6^ Studies have also shown changes in quantity and affinity of β-adrenergic receptors and, perhaps more important, an augmentation in postreceptor signaling cascades ultimately leading to a state of adrenergic hyperresponsiveness.^6,7^

Common precipitants of thyroid storm include infection, surgery, trauma, and radioactive iodine. There are several theories as to how triggers could precipitate thyroid storm. These include sudden decreased affinity of thyroid hormone binding proteins leading to higher levels of active hormone, increased thyroid hormone uptake into cells, and transient loss of central sympathetic nervous system downregulation in the setting of adrenergic hyperresponsiveness.^5,6,8^

## Cocaine and the Thyroid Axis

Cocaine inhibits presynaptic reuptake of biogenic amines.^2^ It is the inhibition of dopamine reuptake that is thought to explain the euphoric and addictive properties of cocaine.^2^ The additional inhibition of norepinephrine reuptake induced by cocaine explains the sympathetic overdrive that is observed in cocaine intoxication. It is possible that this stimulant effect of cocaine intoxication in a patient with the adrenergic hyperresponsiveness associated with thyrotoxicosis could trigger thyroid storm, as exemplified by our patient.

The interplay of cocaine use and thyroid function is complex and poorly understood. Cocaine- and amphetamine-regulated transcript (CART) peptides were first discovered by looking for brain transcripts that were increased following acute cocaine or methamphetamine exposure.^9^ CART peptides have since been shown in rats to innervate hypothalamic neurons and exhibit a stimulatory effect on transcription of thyrotropin-releasing hormone (TRH).^10^ Furthermore, several studies have documented a diminished thyroid-stimulating hormone (TSH) response to TRH in people with concurrent cocaine abuse.^11-13^ After 30 days of abstinence, however, TRH produced an increased TSH response.^13^ A hypothesis for this interaction is that chronic cocaine use could result in a dopamine and norepinephrine-mediated increase in TRH levels that leads to downregulation of TRH receptors and decreased TSH.^11^

Despite these connections between thyroid hormone regulation and cocaine use, acute as well as repeated cocaine stimulation in rats was not shown to produce a change in serum T3 and T4 levels.^14^ Thyroid function tests in 22 heavy cocaine users studied prior to admission to a treatment center showed no statistically significant difference when compared to a control population.^15^ Conversely, in a retrospective study that looked specifically at patients with diagnosed hyperthyroidism (90% subclinical), there was an increased incidence of cocaine use.^16^ It was theorized that an enhanced responsiveness to cocaine’s stimulatory effects could contribute to increased use of the drug.^16^

## Treatment

Benzodiazepines are the mainstay of treatment for cocaine intoxication and have beneficial effects on cocaine-induced chest pain as well as central nervous system disturbances.^2^ The treatment of thyroid storm requires a multistep approach with identification and treatment of any precipitant, as well as treatment aimed at decreasing thyroid hormone production, thyroid hormone release, and the peripheral effects of existing thyroid hormone.^17^

Perhaps the most effective immediate treatment for thyroid storm is blockage of the adrenergic effects created by excess thyroid hormone. This is most often accomplished with high-dose propranolol, a nonselective β-blocker, which also functions to block some peripheral conversion of T4 to T3.^5^ While the frequently cited contraindication of β-blockade in a patient with cocaine use has been reasonably called into question,^18,19^ it was propranolol that was originally shown to increase cocaine-associated coronary vasoconstriction.^20^ Interestingly, there are cases of increased α-adrenergic effects following labetalol administration in cases of pheochromocytoma, raising concerns even about the use of β-blockers with α-blocking action in extreme hyperadrenergic states.^21^ To the best of our knowledge, there exists no data on the safety of the use of β-blockade in patients presenting with a mixed picture of thyroid storm and cocaine intoxication to guide clinical decision making regarding use of selective or nonselective β-blockers.

Thionamides are the preferred agents to block new thyroid hormone production and act via inhibition of thyroid peroxidase. While propylthiouracil (PTU) has less convenient dosing requirements and a higher toxicity profile compared to methimazole, PTU is often preferred in the acute management of thyroid storm because it additionally decreases peripheral conversion of T4 to T3 by as much as 20% to 30% in the acute setting.^22^ Iodine administration acutely acts to block organification of iodide and synthesis and release of thyroid hormone via the Wolff Chaikoff effect. Iodine should typically be administered 30 to 60 minutes after thionamide therapy has been started to prevent stimulation of new hormone synthesis. Hydrocortisone administration is also recommended by current guidelines and treats possible relative adrenal insufficiency while also decreasing T4 to T3 conversion.^23^

## Conclusion

The case of Mr S highlights the importance of developing a broad differential diagnosis to prevent premature closure. Our patient presented with a reported cocaine binge and displayed many signs and symptoms that could be directly explained by cocaine intoxication alone. Other diagnoses to consider in a patient with substance abuse, tachycardia, and fever include infectious etiologies, such as pneumonia or endocarditis, or pulmonary embolus. A thorough history and physical examination prompted consideration of thyrotoxicosis as an alternative explanation for his historical weight loss and diarrhea as well as his current fever, tachycardia, and mental status changes. The high morbidity and mortality associated with thyroid storm necessitates that clinicians consider this diagnosis in the differential. Mr S was promptly diagnosed and treated for thyroid storm as well as cocaine intoxication and ultimately received more definitive treatment for his Graves’ disease.
